# Extracellular PKM2 Repairs Colon Damages in Colitis Model by Regulating Macrophage Polarity

**DOI:** 10.3390/ijms27219853

**Published:** 2026-11-03

**Authors:** Bin Li, Guangda Peng, Naseer A. Kutchy, Siva C. Chabattula, Jenny J. Yang, Zhi-Ren Liu

**Affiliations:** 1 Division of Hematology and Oncology, School of Medicine, The University of Alabama at Birmingham, Birmingham, AL 35233, USAnakutchy@uabmc.edu (N.A.K.);; 2 Department of Biology, Georgia State University, Atlanta, GA 30303, USA

**Keywords:** pyruvate kinase M2, colitis, macrophage, integrin α_v_β_3_, arginase1, glycolysis

## Abstract

Inflammatory immunity exacerbates tissue damage in the tissue injury. Resolution of the inflammation trigger tissue repair and regeneration. We report here that the extracellular PKM2 (EcPKM2) modulates immunity at colon damage site by facilitating M2 macrophage polarization in response to Dextran Sodium Sulfate (DSS)-induced damage. The effects of EcPKM2 on macrophage polarity led to decreased inflammation and facilitated the repairment of colon damages due to a reduction in epithelial cell death in a DSS-induced Ulcerative colitis mouse model. Interestingly, EcPKM2 facilitates intestinal mucosal lining repair by promoting colon epithelial proliferation. EcPKM2 protects intestinal epithelial cells from death and promotes the epithelial cell proliferation by promoting M2 macrophages’ polarity. Our study suggests a potential new therapeutic strategy by targeting macrophage polarity for Ulcerative Colitis treatment.

## 1. Introduction

Ulcerative Colitis (UC) is an inflammatory bowel disease (IBD) that affects a large population worldwide. UC primarily affects the large intestine with confinement to the mucosal layer of the intestine. The pathogenesis of UC is complex and is not well understood. It is generally believed that UC results from environmental exposures to hazards in populations with a genetic predisposition. Epithelial barrier disruption, dysregulated tissue immune responses, and dysbiosis are all key players in initiating and perpetuating inflammation in UC development. A typical characteristic of UC is the presence of continuous colonic inflammation. Cells of both innate and adaptive immunity play a critical role in UC development and progression [1]. Among all immune cells involved, macrophages are one of the most abundant leukocyte cell types found in the luminal gastrointestinal mucosa. Macrophages act like a gatekeeper for intestine immune responses to mucosal damage and subsequent homeostasis [2]. Furthermore, macrophages provide the healing power to drive mucosal healing in response to broken mucosal barriers in UC. In response to the colonic damage and inflammatory cytokines that secrete during UC development, macrophages are differentiated into M1 polarity, which enacts the functions of macrophages in intestinal inflammation and gut barrier destruction [3]. During intestinal tissue homeostasis, macrophages are polarized to M2 macrophages. The M2 macrophages activate Wnt-signaling, which promotes mucosal repair. Mucosal healing is the critical initiator for sustained drive force for UC remission. Thus, target macrophage polarization is recognized as a potentially effective strategy in treatment of UC [4].

Pyruvate kinase, an enzyme that catalyzes the last reaction in glycolysis, has four isoforms, L/R and M1/M2. Different isoforms are expressed in different tissue types or under different physiological conditions. PKM2 can form a homodimer or a homotetramer. The tetramer is active as a pyruvate kinase [5,6], while the dimer can be a protein kinase [7,8,9]. Interestingly, recent studies demonstrate that PKM2 is functionally involved in multiple cellular processes in different cellular locations, including metabolism control, transcription regulation, and chromatin packaging [8,10,11]. PKM2 has long been observed in patients with solid tumors and various inflammation diseases recording high levels [12,13,14]. Our previous studies demonstrated that EcPKM2 facilitates tumor growth by promoting angiogenesis [15]. During cutaneous wound repair, EcPKM2 released by infiltration neutrophils facilitates wound repair at the wound site [16]. We recently reported that PKM2 is released by myofibroblasts, and secreted EcPKM2 facilitates fibrogenesis [17]. EcPKM2 also facilitates cancer progression by modulating tumor immunity. EcPKM2 promotes alternative activated macrophages (M2) in tumors, thus favoring suppression of tumor immunity. EcPKM2 promotes M2 macrophages by interacting with integrin α_v_β_3_ on the macrophage and consequently activating the integrin FAK–PI3K signaling axis [18]. In the present report, we demonstrate that EcPKM2 repairs DSS-induced colon damage by modulating inflammation at the UC site. EcPKM2 preserved the colon mucosal structure in the DSS-induced UC model by preventing colonic epithelial from apoptosis. EcPKM2 facilitates angiogenesis and promotes M2 macrophages in a mouse model of DSS-induced UC. EcPKM2 facilitates mucosa epithelial proliferation at the UC site by promoting M2 macrophages. Our study uncovers an important mechanism of EcPKM2 in the protection of intestinal mucosal barriers in the DSS-induced UC mouse model. Our study suggests a potential new therapeutic strategy by targeting macrophage polarity for Ulcerative Colitis treatment.

## 2. Results

EcPKM2 facilitates the repairment of colon damage in a DSS-induced UC mouse model.

**Figure 1:**
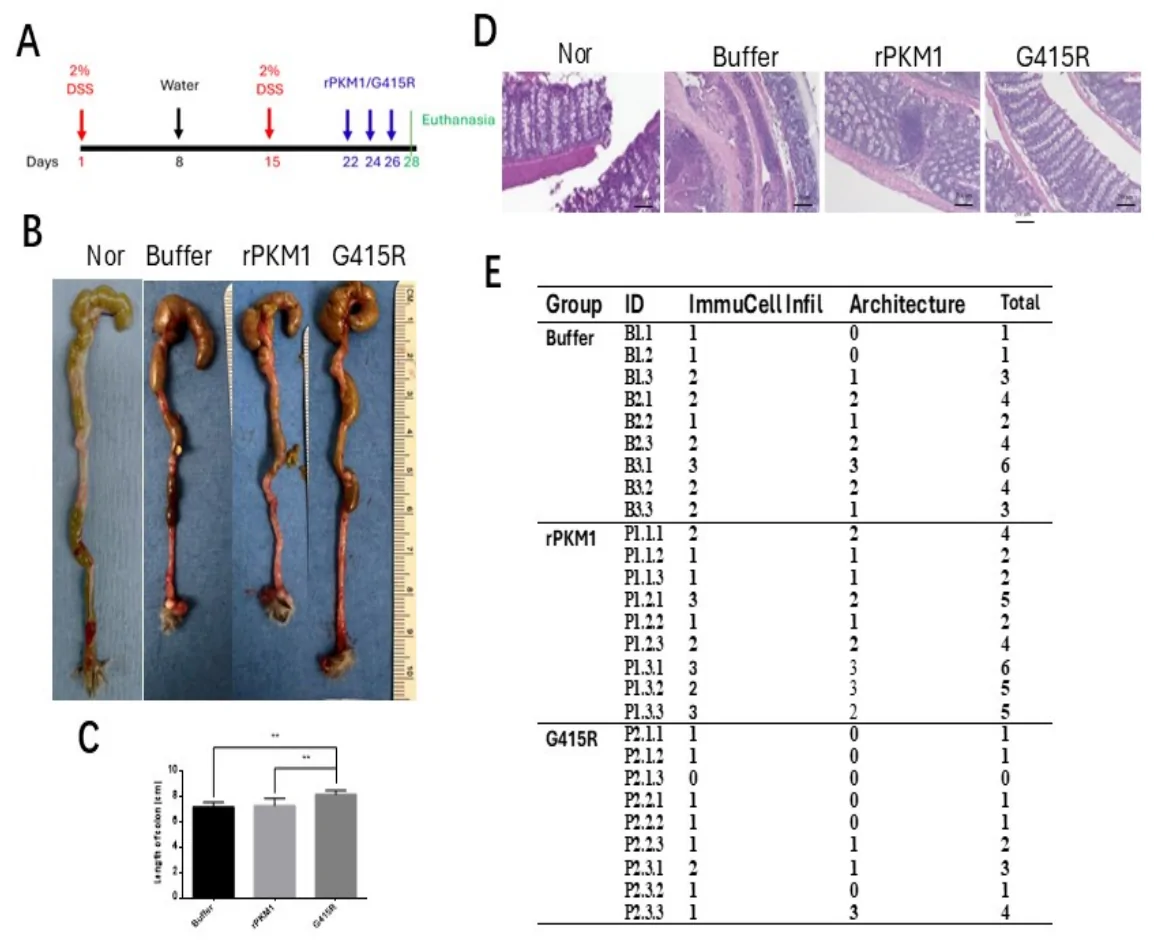
EcPKM2 protects colon from damage in DSS-induced UC mouse models. (**A**) Schematic illustration of experimental design for DSS-induced mouse UC model and subsequent treatments. (**B**) Representative images of gross morphology of colons isolated from the indicated groups (Group size *n* = 7) at the experimental endpoint. (**C**) Quantification of colon lengths from the indicated groups. (**D**) Representative images of hematoxylin and eosin (H&E) staining of colon tissue section. Scale bar 200 μm. (**E**) Histological scores, inflammation cell infiltration (ImmuCell Infil), intestinal architecture damage (Architecture), and total scores (Total) of colon sections from the indicated groups. Nor in (**B**,**D**) are the samples from normal healthy mice (without DSS treatment). Error bars in (**C**) represent mean ± SEM. Statistical significance was determined by one-way ANOVA, ** *p* < 0.01.

EcPKM2 plays an important role in modulating inflammation and facilitation of tissue repair [16]. UC is a disease condition where inflammatory responses cause tissue damage in the colon. We thus questioned whether EcPKM2 would have any role in UC progression. To this end, we utilized an established DSS-induced UC mouse model. Ten-week-old C57BL/6 mice were fed with 2% (*w*/*v*) DSS solution as drinking water in the first and the third week. The mice were fed with regular drinking water in the weeks between the DSS feeding. The mice were treated with recombinant G415R, a mutant of PKM2, recombinant PKM1 (rPKM1), and buffer QOD in three doses (Figure 1A). The rPKM1 is a comparison reference for PKM2, while G415R was used due to its functional equivalence to dimer PKM2 and higher solubility of the mutant for in vivo experiments. The animals were euthanized 2 days after the last G415R treatment dose. We assessed the effects of G415R on colon damage caused by DSS. Due to mice body weights being largely stable and rectal bleeding almost stopping after the second round of DSS, the subsequent treatments did not change the mouse body weight or rectal bleeding. Thus, the typical disease activity index is not reported here. Measurement of colon lengths indicated that the G415R treatment recovered the DSS-induced colon damage as compared to those of rPKM1 and the buffer groups (Figure 1B,C). To further explore the impact of EcPKM2 on damage in the colons of UC mice, histological and morphological changes in the intestinal tissues were examined using H&E staining. Histological analysis revealed significant pathological changes in the colonic tissues of the DSS-induced UC mice, including the disappearance of a significant portion of goblet cells, disarray of intestinal glands, alteration in crypt structure, infiltration of inflammatory cells into the mucosal and submucosal layers, and edema in the colonic muscular layer. G415R but not rPKM1 nor the buffer group largely recovered the pathological damage caused by DSS (Figure 1D). Histological examination of inflammatory cell infiltration and intestinal anatomic architecture (via a standardized scoring system [19]) suggested that G415R treatment significantly decreased the scores of both inflammatory cell infiltration and intestinal anatomic architecture compared to the buffer and rPKM1 groups (Figure 1E). Histological analysis was also performed with colonic tissues of the same age of healthy normal B6 mice. There was no inflammatory cell infiltration nor abnormality in the intestinal anatomic architecture (Score 0,). Our results indicate that EcPKM2 facilitates the recovery of intestinal damage in the DSS-induced UC mouse model.

The Effects of EcPKM2 on Fibrosis and Angiogenesis in the UC Areas.

**Figure 2:**
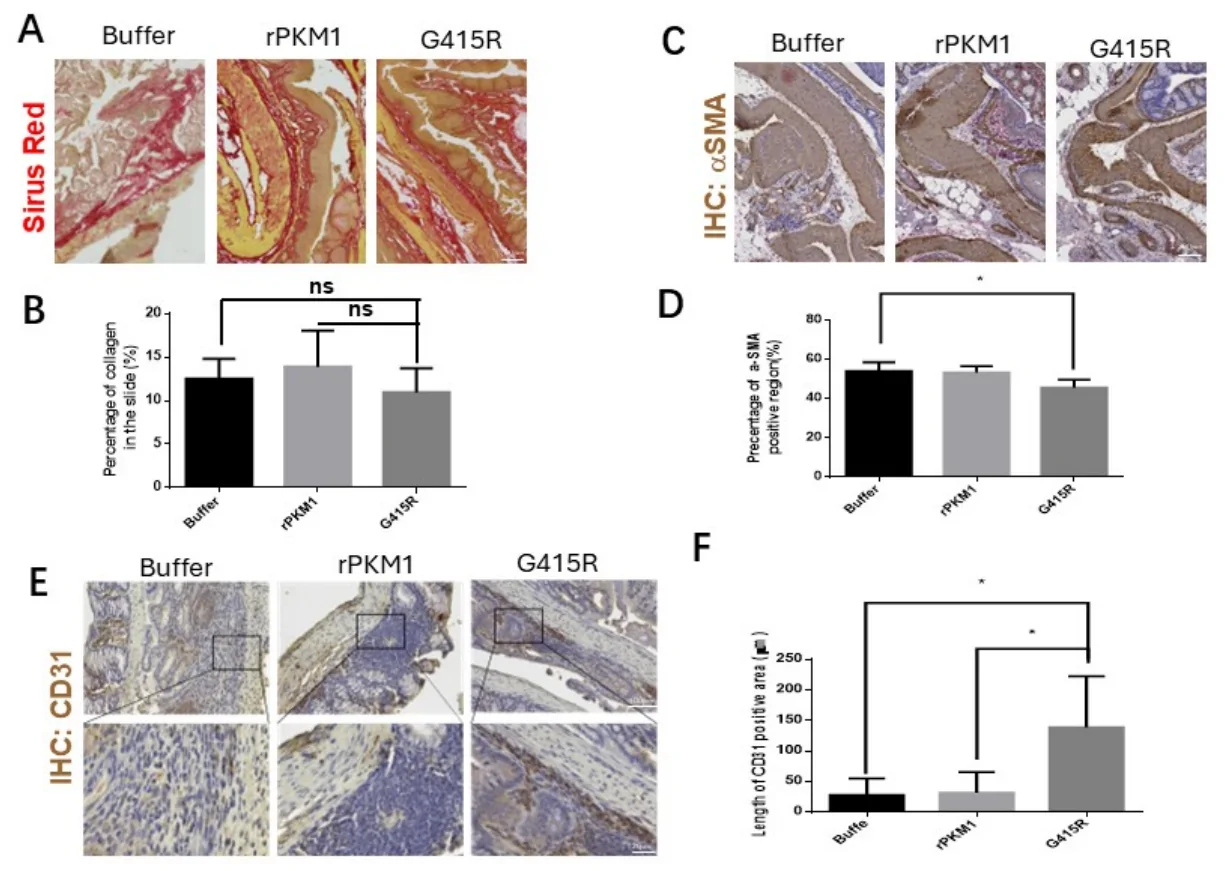
EcPKM2 modulates angiogenesis and fibrosis in the UC areas. (**A**,**B**) Representative images (**A**) and quantification (**B**) of Sirius Red staining (collagen) of colonic sections from the indicated groups. Results are presented as percentage of collagen areas in section. (**C**,**D**) Representative images (**C**) and quantification of IHC staining of aSMA of colonic sections from the indicated groups. Results are presented as percentage of aSMA positive regions. (**E**,**F**) Representative images (**E**) and quantification (**F**) of IHC staining of CD31 of colonic sections from the indicated groups. Results are presented as length of CD31 positive in mm. The quantification is statistical mean of 3 randomly selected views fields in 3 randomly selected sections per animal. Scale bars in (**A**,**C**) 200 mm, (**E**) 100 mm, 20 mm in the enlarged images of (**E**). Error bars in (**B**,**D**,**F**) represent mean ± SEM. Statistical significance was determined by one-way ANOVA. * *p* < 0.05, ns *p* > 0.05.

To understand how EcPKM2 facilitates the recovery of DSS-caused intestinal damage, we examined the effects of G415R on the accumulation of collagen fibers and fibroblast activation. Sinus Red staining revealed that EcPKM2 led to slightly reduced accumulation of collagen fibers in the UC areas (Figure 2A,B). This is consistent with IHC staining of aSMA showing less aSMA-positive cells in the UC areas (Figure 2C,D). We previously reported that EcPKM2 promotes angiogenesis in wound repair and in tumor progression [15,16]. We therefore reasoned that G415R might affect angiogenesis in the UC areas. CD31 staining demonstrated that G415 increased CD31 staining compared to the rPKM1 and buffer groups (Figure 2E,F). Although the increase in CD31 staining does not prove the effects of G415R in colon angiogenesis, the potential role of G415 in increases in angiogenesis indicates a possible function of the protein in promoting initiation of damage repair.

EcPKM2 Modulates Immunity and Regulates Macrophage Polarity in the UC Areas.

**Figure 3:**
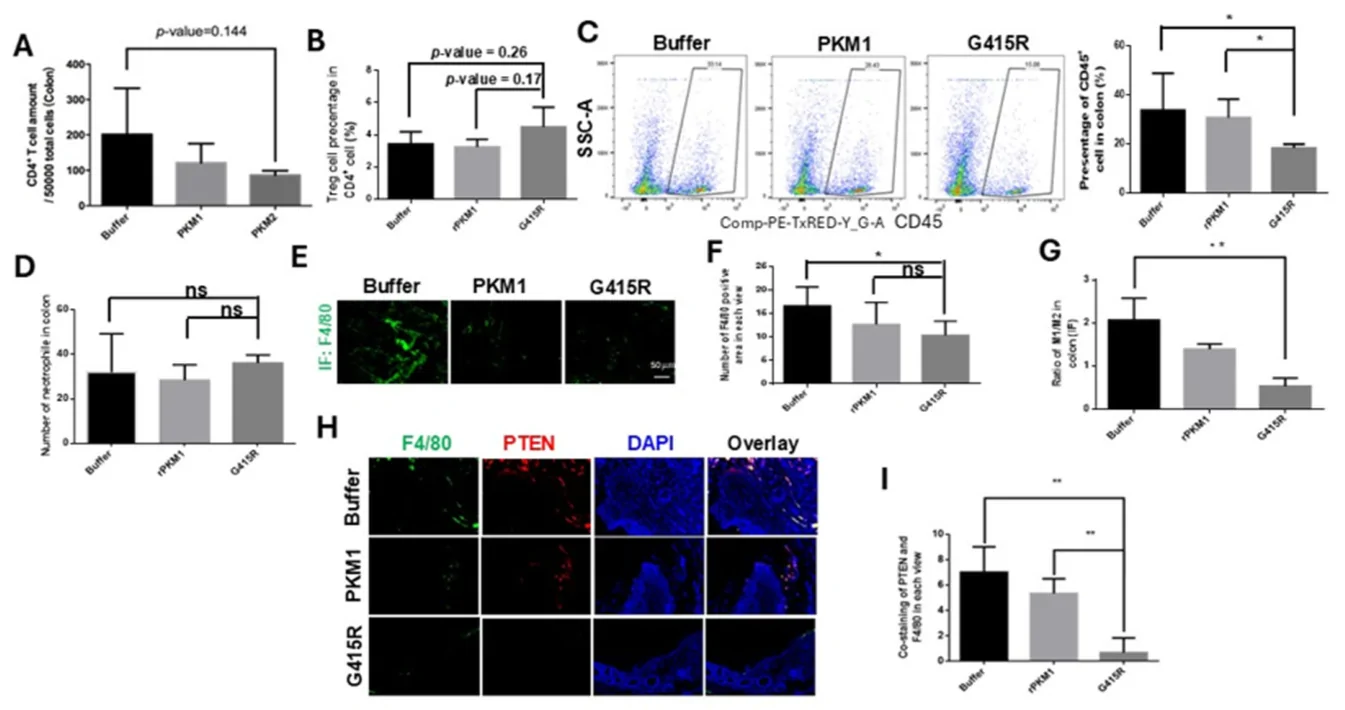
EcPKM2 modulates immunity and regulates macrophage polarity in the UC mice. (**A**–**D**) CD4^+^ cells ((**A**), cell # in total 50,000 cells), Treg cells ((**B**), percentage in total CD4^+^ cells), CD45^+^ cells ((**C**), right panel, percentage in total colon cells), and neutrophils ((**D**), cell # in total 10,000 cells of colon tissue) in UC areas of colon of DSS-induced UC mice treated by indicated agents were measured by flow cytometry. Left panel in C shows example plots of FACS analyses of CD45^+^ cells in UC colon tissues. (**E**,**F**) Representative images (**E**) and quantification (**F**) of IF staining of F4/80 of colonic sections from the indicated groups. Scale bar in (**E**) 50 μm. Quantifications are presented as number (percentage) of F4/80 positive areas per view field. (**G**) Quantification of M1/M2 macrophage ratio measured by IF staining and flow cytometry in colonic tissues from the indicated groups. (**H**,**I**) Representative images (**H**) and quantification (**I**) of co-staining of F4/80 and PTEN by IF staining in colonic tissues from the indicated groups. Error bars in (**A**–**I**) represent mean ± SEM. from 7 mice. Statistical significance was determined using one-way ANOVA. ns *p* > 0.05, * *p* < 0.05, ** *p* < 0.01.

EcPKM2 promotes wound repair by suppressing inflammation at the wound site [16]. We thus questioned whether EcPKM2 modulates immunity in the UC damaged areas. To this end, we analyzed the effects of G415R on immune cells populations in UC areas. Firstly, a minor decrease in total CD4^+^ T cells (Figure 3A) along with a minor increase in Regulatory T cells (Tregs) (Figure 3B and Figure S1A) was observed in the G415R treated group compared to the rPKM1 and buffer groups, indicating a state of inflammation suppression induced by G415R. This is consistent with observation that G415R treatment led to a minor decrease in CD45^+^ leukocytes (Figure 3C). There was no significant change in neutrophils among the three treatment groups (Figure 3D and Figure S1B). We previously observed the effects of EcPKM2 in regulating macrophage polarity in tumors [18]. Thus, we probed whether G415R regulated macrophage levels and polarity in DSS-induced UC model. IF staining of F4/80 a macrophage marker suggested a modest decrease in macrophage at the UC area of G415R-treated UC mice compared to the rPKM1 and buffer groups (Figure 3E,F). The observation is consistent with the observed minor decrease in CD45^+^ leukocytes in the UC area (see Figure 3C). In consistency with our previous observation, iNOS and CD206 IF staining suggested that G415R led to a decrease in M1 macrophages and an increase in M2 macrophages (Figure S2). There was a dramatic decrease in the M1/M2 ratio in the UC areas compared to the rPKM1 and buffer groups (Figure 3G and Figure S2). The observations were also confirmed with FACS analysis (Figure S3A,B). Clearly, a decrease in the M1/M2 macrophage ratio is consistent with the role of G415R in reducing tissue destructive inflammation in the intestine of the UC colon. Our previous studies showed that EcPKM2 regulated macrophage polarity by FAK-PI3K signaling axis, which subsequently controlled PTEN expression in macrophages [18]. To understand whether G415R acts via a similar mechanism here in the UC model, we probed the PTEN expression in the macrophages. G415R treatment led to significantly less F4/80 and PTEN co-staining (Figure 3H,I). Our observation is consistent with previous studies in other laboratories that PTEN expression in macrophages promotes macrophage polarity M2 to M1 and inhibits macrophage polarization from M1 to M2 [20].

EcPKM2 recovers intestinal mucosal epithelial and facilitates mucosal barrier repair.

**Figure 4:**
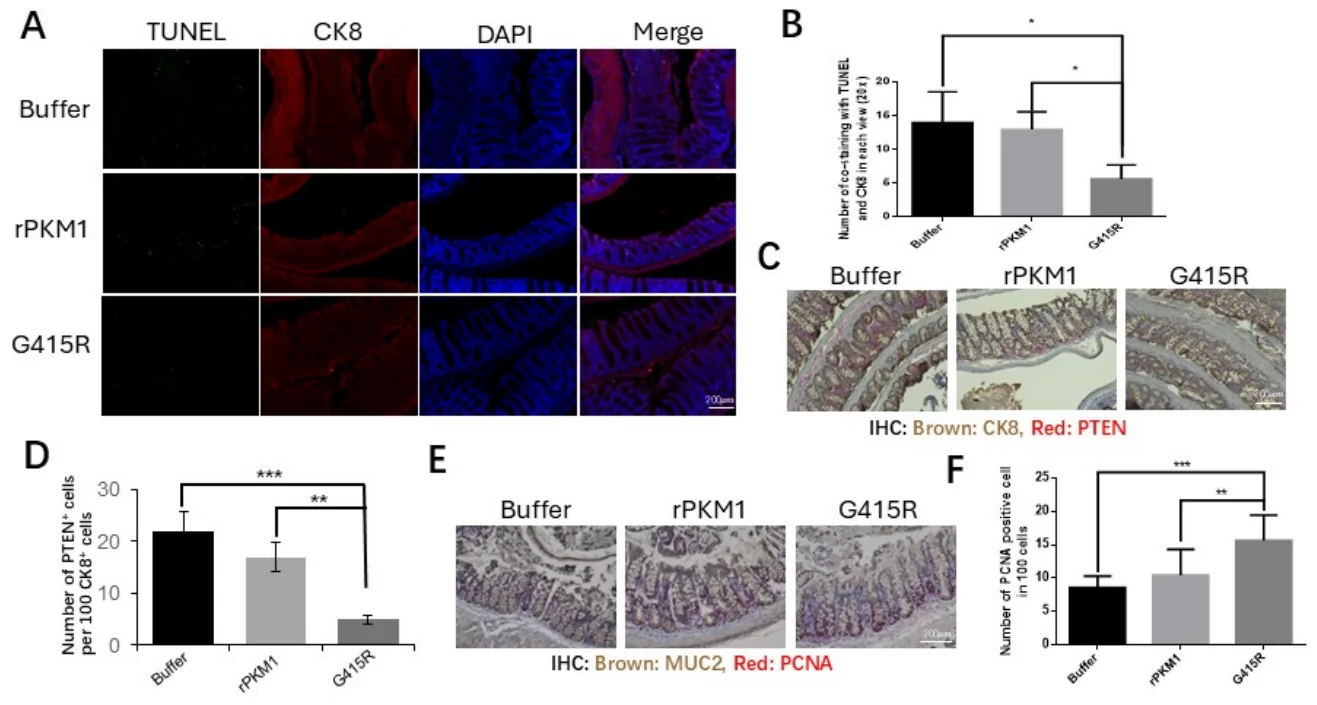
EcPKM2 recovers intestinal mucosal epithelial and facilitates mucosal barrier repair. (**A**,**B**) Representative images (**A**) and quantification (**B**) of IF staining of TUNEL (green) and CK8 (red) in intestinal tissue sections from UC areas of the mice treated with indicated agents. Nuclei were counterstained with DAPI (blue). Merged images are overlay of different staining. Quantifications in (**B**) are presented as number of both CK8 and TUNEL positive cells per view field. (**C**,**D**) Representative images (**C**) and quantification (**D**) of IHC staining of CK8 (brown) and PTEN (red) in intestinal tissue sections from UC areas of the mice treated with indicated agents. Quantifications in (**D**) are presented as number of PTEN positive cells in 100 CK8 positive (PTEN^+^/CK8^+^). (**E**,**F**) Representative images (**E**) and quantification (**F**) of IHC staining of MUC2 (brown) and PCNA (Red) in intestinal tissue sections from UC areas of the mice treated with indicated agents. Quantifications in (**F**) are presented as number of PCNA positive cells in 100 MUC2 positive (PCNA^+^/MUC2^+^). Scale bars in (**A**,**C**,**E**) 200 μm. Error bars in (**B**,**D**,**F**) represent mean ± SEM. Statistical significance was determined by one-way ANOVA. * *p* < 0.05, ** *p* < 0.01, *** *p* < 0.005.

A unique feature in the G415R treatment group, comparing to the rPKM1 and buffer groups, is the preservation of the mucosal structure, indicating a potential role of G415R in the recovery of intestinal mucosal lining resulting from DSS-induced damage. The mucosal epithelial lining is the key component of the mucosal barrier [21]. We therefore examined the effects of G415R on the intestinal mucosal epithelial lining in the UC areas. TUNEL and CK8, an epithelial cell marker, co-staining demonstrated that G415R treatment led to less mucosal epithelial cell death (Figure 4A,B). Since G415R decreased PTEN in the macrophages, we asked whether G415R also decreased PTEN in the mucosal epithelial lining that therefore protect the epithelial cells from death. IHC staining revealed that PTEN did not co-stain with CK8 (Figure 4C,D). M2 macrophages play important role in repairing mucosal barriers in UC by promoting mucosal epithelial proliferation [22]. We reasoned whether EcPKM2 also promotes mucosal epithelial proliferation in the DSS-induced UC model. We therefore examined epithelial proliferation by co-staining of MUC-2, a marker of intestinal epithelial and mucosal barriers [23], and PCNA. Clearly, G415R resulted higher levels of MUC-2 and PCNA co-staining than those of the rPKM1 and buffer groups (Figure 4E,F). Our experiments suggest that G415R facilitates mucosal repair by protecting mucosal epithelial from death and promoting the epithelial proliferation.

EcPKM2 does not protect cultured colon epithelial cells from apoptosis and does not promote cultured colon epithelial cells proliferation.

**Figure 5:**
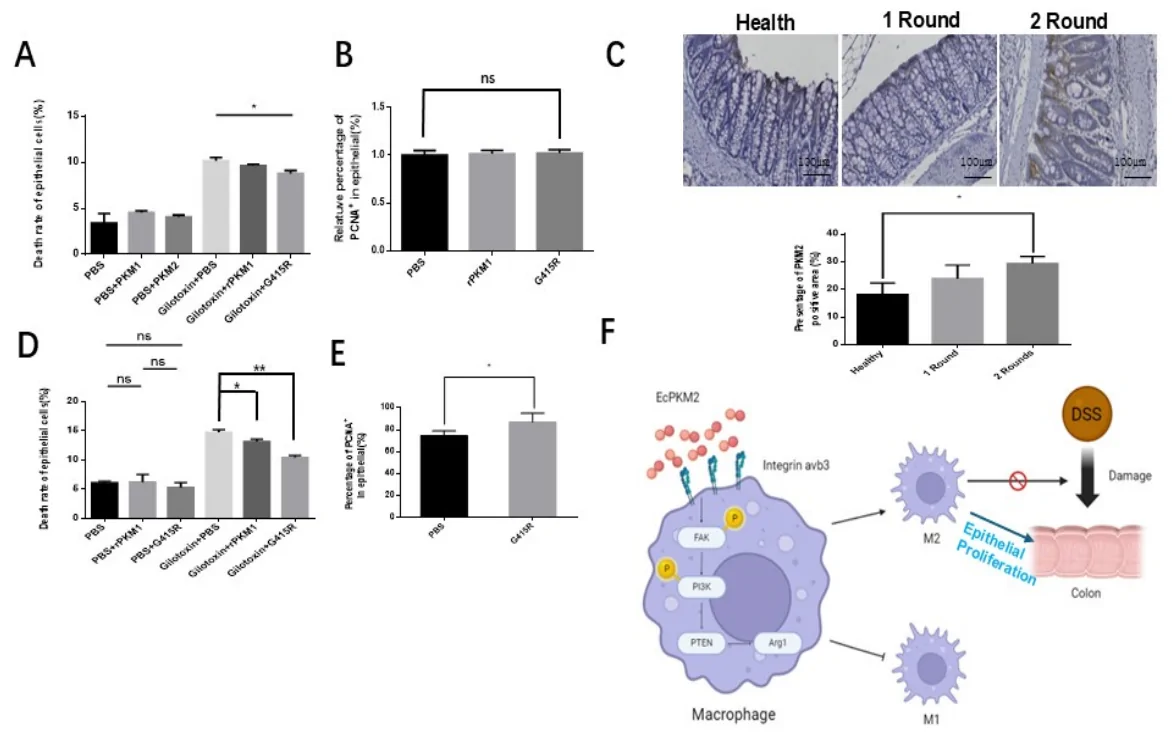
EcPKM2 protects cultured colon epithelial cells from apoptosis and promotes cultured colon epithelial cells proliferation by M2 macrophage polarization. (**A**,**D**) Quantitative analysis of mouse primary colon epithelial cells (mPCE) death rate (TUNEL) under the treatment with indicated agents. The mPCE cells were co-cultured (**D**) or without co-culture (**A**) with RAW246.7 cells that were pretreated with the indicated agents. The results are presented as death rate of mPCE epithelial cells (%). (**B**,**E**) Quantification of mPCE cell proliferation (PCNA positive) under the treatment with indicated agents. The mPCE cells were co-cultured (**E**) or without co-culture (**B**) with RAW246.7 cells that were pretreated with the indicated agents. The results are presented as percentage of PCNA positive in epithelial cells (%) in (**E**) and relative percentage of PCNA positive in epithelial (%) by set the buffer treated cells as 1 (**B**). (**C**) Representative images (top) and quantification (bottom) of IHC staining of PKM2 at different time points before and after DSS administration. Scale bars in (**C**) is 100 μm. PKM2 levels in colon tissues of DSS-induced UC mice were measured by IHC staining of PKM2 at different time points before and after DSS administration. The PKM2 levels are presented as percentage of PKM2^+^ staining per view field. (**F**) Schematic illustration of actions of EcPKM2 in DSS-induced mouse UC model. Error bars in (**A**–**E**) represent mean ± SEM. Statistical significance was determined by one-way ANOVA. ns *p* > 0.05, * *p* < 0.05, ** *p* < 0.01.

How does EcPKM2 protect intestinal mucosal epithelial cells from death, and how does EcPKM2 promote the epithelial cells proliferation in the UC areas are open questions. We questioned whether EcPKM2 exerts the effects directly on epithelial cells. To answer the question, we employed primary colon epithelial cells (mPCE) from C57BL/6 mice. Gliotoxin mechanistically mimics the toxic effects of DSS on intestinal epithelial [24]. We examined the effects of G415R on the activity of gliotoxin in induction of the death of mPCE cells. Evidently, G415R had minor effects in preventing the mPCE cells from gliotoxin-induced apoptosis (Figure 5A). G415R treatment led to intestinal epithelial proliferation in the DSS-induced UC mice (see Figure 4E,F). We therefore analyzed whether G415R would directly promote mPCE cells proliferation. Clearly, addition of G415R to the culture medium of mPCE cells did not lead to cell proliferation (Figure 5B). We conclude from our experiments that EcPKM2 does not directly protect cultured colon epithelial cells from apoptosis and does not directly promote cultured colon epithelial cells proliferation.

## 3. Discussion

Our study in this report uncovers an important role of EcPKM2 in the protection of intestinal mucosal barriers in a DSS-induced UC mouse model. EcPKM2 protects colon tissue from damage and facilitates tissue repair by modulating immunity in the damaged areas of the UC mice. Our observation provides another example demonstrating that EcPKM2 protects tissue from damage and facilitates wound repair by promoting angiogenesis and suppressing inflammatory immunity, which consequently reduces tissue damage. Similar to our previous observation in tumors, EcPKM2 also regulates macrophage polarity in the tissue damage site of the UC mice. Our results clearly support our previously proposed theme that EcPKM2 may bridge the inflammatory response to a later proliferation/regeneration phase (fibrogenesis/angiogenesis) during tissue injury repair and cancer progression. Herein, we are showing another example that supports our hypothesis.

EcPKM2 is released from neutrophils in cutaneous wounds and cancer cells in tumors [15,16]. We probed the endogenous PKM2 in UC tissues samples by IHC staining of PKM2. PKM2 was detected in very low levels in the healthy colon tissue. PKM2 levels were significantly increased in colon tissues upon each round (week) of DSS treatment in the UC mice (Figure 5C). A similar pattern was observed in our previous studies of wound repair of cutaneous wounds and myocardial infarction. PKM2 is released to the wound site 3 days after wound induction [16], while PKM2 is expressed, and EcPKM2 is released 4 days after infarction induction [25]. This pattern may suggest a possible common intrinsic mechanism of role of EcPKM2 in tissue injury protection and repair. However, the expression and extracellular release of PKM2 occur too late and at insufficient levels to effectively protect against early tissue damage during the initial stages of UC induction in intestinal tissue. Exogenous introduction of PKM2 may fulfill the function of EcPKM2 in tissue injury protection in due time. Where does EcPKM2 come from? Our studies have demonstrated that EcPKM2 can be expressed and secreted by several cell types, e.g., cancer cells, neutrophiles, myofibroblasts, and cardiomyocytes [15,16,17,25]. One possible source of EcPKM2 is infiltrating neutrophils. High neutrophils levels are presented at damaged tissue sites in UC, particularly at early time points [26]. However, the possibility that EcPKM2 is secreted by other cell types cannot be ruled out.

G415R treatment protected the intestinal mucosal lining from damage. The treatment also facilitated mucosal repair. Interestingly, G415R treatment protected intestinal epithelial cells from death and promoted epithelial proliferation in the intestinal tissue of DSS-induced UC mice. An open question is how EcPKM2 exerts its actions on intestinal epithelial cells. Clearly, our experiments with cultured colon epithelial cells showed that G415R did not have effects on protecting epithelial cell apoptosis under gliotoxin treatment. G415R also did not promote cultured epithelial cell proliferation. Since major colon tissue damage is caused by inflammatory responses in the UC [27], it is conceivable that G415R protected epithelial cells from death by reducing inflammation in the colon in the UC model. We further asked whether EcPKM2 protected colon epithelial cell apoptosis by modulating macrophage polarity. EcPKM2 co-culture with Raw246.7 cells significantly protected mPCE from gliotoxin-induced apoptosis (Figure 5D). It is well documented that macrophage polarity, M2 macrophages, promote intestinal mucosal epithelial cell proliferation, thus facilitating mucosal barriers repair in UC [28]. G415R promoted M2 macrophages at the damaged site of UC. Thus, our logic assumption is that EcPKM2 promotes M2 macrophages in the UC, which therefore facilitates intestinal mucosal epithelial cell proliferation. We tested the colon epithelial cell proliferation in co-cultured RAW246.7 cells. Pre-treatment of RAW246.7 cells with G415R before co-culture resulted in a higher proliferation rate of mPCE. The proliferation of the epithelial cells was not observed with pre-treatment of the RAW246.7 cells with a buffer (Figure 5E). The observation provided support for our assumption the EcPKM2 facilitates intestinal mucosal epithelial cell proliferation indirectly by regulating M2 macrophage polarity (Figure 5F). We previously observed that EcPKM2 facilitates fibrogenesis in cutaneous wound repair [16], liver and lung fibrosis models [17], as well as in tumors [15]. However, we did not observe such effects of EcPKM2 here in the DSS-induced UC model. In fact, EcPKM2 exerted opposite effects and slightly decreasing intestine fibrosis. It is traditionally believed that UC, unlike Crohn’s Disease, is a non-fibrotic mucosal disease. More recent studies revealed that fibrosis also occurs in the mucosal and submucosal layers of UC. Chronic and cyclical injuries of the epithelial mucosa activate fibroblasts and drive intestinal mucosa fibrosis [29,30]. Since EcPKM2 prevented mucosa epithelial proliferation in the UC damage, it is possible that the protection effects of EcPKM2 prevented mucosa fibroblasts activation and consequentially resulted in less fibrosis. Similar effects of EcPKM2 were also observed in the effects of EcPKM2 in myocardial infarction where EcPKM2 protect cardiomyocytes from death, thus prevented activation of cardiac fibroblasts during infarction [25]. Although we do not fully understand the mechanism underlines this effect, the effects of EcPKM2 in reducing intestine fibrosis would be beneficial in the treatment and management of UC, as intestinal fibrosis is an important marker of the severity of disease progression [30].

Most current strategies in treatment of UC are focused on inhibiting inflammatory immune activity achieving by blocking various inflammatory signaling such as the number of cytokine activities [31]. One drawback is that such an approach often leads to unwanted immunosuppression. An important implication of our observation is the potential of using EcPKM2 as a treatment drug for patients with UC. The dual action of EcPKM2 in promoting angiogenesis and regulation of macrophage polarity will provide important advantages as the mechanism of drug action in treatment of UC. As EcPKM2 is presented in blood circulation in various levels in both healthy individuals and individuals with various inflammatory diseases, it is speculated that the administration of EcPKM2 may be relatively safe and well tolerated. Our preliminary studies showed that G415R is not toxic to healthy mice at doses as high as 50 mg/kg Q.O.D for eight doses. Of course, more extensive toxicology studies are required for developing G415R as a treatment drug for UC. Since two cycles of DSS induction may not mimic the chronic nature of UC patients, one major limitation of our study is that the validation of the effectiveness of G415R as a potential treatment agent for UC is currently limited.

## 4. Methods

### 4.1. Recombinant Protein Expression and Purification

The cDNAs that encode human PKM1 and PKM2 mutant G415R were purchased from Adgenes. The cDNAs were subcloned into bacterial expression vectors. The recombinant proteins were expressed and purified from bacterial lysates by a two-column procedure, as in previous reports [8,15].

### 4.2. Cell Line and Mouse Bone Marrow Derived Macrophages

Cell lines: Cell line Raw264.7 was purchased from *ATCC*. Mouse colonic primary epithelium cell (mPCE) was purchased from Cell Biologics. Cells were cultured according to vendor’s instruction.

### 4.3. Animal Model and Treatments

All animal experiments conformed to the guidelines of the NIH Guide for the Care and Use of Laboratory Animals and were approved by the IACUC of The University of Alabama at Birmingham and Georgia State University. At the end of each experiment, the mice were euthanized by CO_2_ inhalation. Animal euthanasia conformed to the guidelines of the NIH Guide for the Care and Use of Laboratory Animals. Animal models: In DSS model, 10 weeks old male C57BL/6 mice are treated with oral-intake 2% DSS solution (*w*/*v*) as the only water resource in this experiment. DSS solution as drinking water was given by 2 rounds of one week in intervals of one week of replacing of DSS solution with normal drinking water [32]. Treatments: After 3 weeks of UC induction by DSS, the animals were treated by 5 mg/kg G415R, rPKM1, or Buffer via i.p. injection QOD for 3 doses. The treatment dose was chosen based on our previous studies [15,17,18,25]. The mice were euthanized 2 days after the last dose treatment. The tissue and other samples were collected for subsequent analysis.

UC standardized scoring system for UC animal model study

Evaluation of colon tissue structural changes and inflammatory cell infiltration was following the standardized grading system via histological scoring. The process strictly follows guidelines published in 2014 [19]. Briefly, the intestinal architecture damage and inflammatory cell infiltration were scored as follows. For intestinal architecture damage, a score of 0 is assigned when the epithelium is intact and the mucosal structure is normal. Focal erosions are scored as 1. Erosions or focal ulceration are scored as 2. Extended ulceration, granulation tissue, or pseudopolyps are scored as 3. For inflammatory cell infiltration, no obvious inflammatory cell infiltration is scored as 0. Inflammatory cell infiltration in the mucosal layer is scored as 1. Inflammatory cell infiltration in both the mucosal and submucosal layers is scored as 2. Transmural infiltration is scored as 3. Total histological score = Inflammatory cell infiltration score + Intestinal architecture damage score. The scores were evaluated from randomly selected three animals/sections and three randomly selected view fields from each section.

FACS sorting macrophages and other immune cells from tissue samples: Briefly, tissue was sliced out from the mice and minced (1 × 1 mm) and digested with collagenase D. After digestion and filtration, the recovered cells were resuspended and washed with FACS staining buffer. The resultant cells were subjected to FACS sorting by various FACS markers indicated in figure legends. The gating strategies for FACS analyses of different cells: CD45 lymphocyte—CD45^+^, CD4 T-cells—CD45^+^/CD3^+^/ CD4^+^, Treg Cells—CD45^+^/CD3^+^/CD4^+^/CD25^+^, M2 macrophages—CD45^+^/F4/80^+^/CD206^+^, M1 macrophages—CD45^+^/F4/80^+^/CD206^−^/iNOS^+^, Neutrophils—CD45^+^/Ly6G^+^.

M1/M2 ratio calculation: In the IF staining analysis, the M1/M2 ratio was calculated by the ratio of iNOS positive staining dividing CD206 positive staining. In the FACS analysis, M1/M2 ratio was calculated by dividing CD206 negative cells by CD206 positive cells within the f4/80 positive cell population.

The procedures for cell apoptosis/viability and proliferation assays and flow cytometry were adopted from our previous reports [15,16,33,34].

Tissue section and cell staining

H&E, IHC, and IF: H&E, IHC, and IF staining procedures were similar to those of previous reports [16,33].

Statistical calculations

Statistical analyses were carried out using the GraphPad Prism 6.0 software. All in vitro experiments were carried out at least 5 times. Group sizes of animal experiments are indicated in figure legends. For image quantifications, FACS, and other analyses, statistical significance was assayed by one way ANOVA. In all figures, * *p* < 0.05; ** *p* < 0.01, *** *p* < 0.001; ns denotes not significant. All data are presented as mean ± SEM.

## Data Availability

The data presented in this study are available upon request from the corresponding author due to potential commercial interests in portions of the data and practical constraints.

## Supplementary Materials

The following supporting information can be downloaded at: https://www.mdpi.com/article/10.3390/ijms27219853/s1.
