# Supplementary material for: Extracellular PKM2 Repairs Colon Damages in Colitis Model by Regulating Macrophage Polarity
Source: Int J Mol Sci. 2026 Nov 3;27(21):9853. doi: 10.3390/ijms27219853 (PMC13764363; doi:10.3390/ijms27219853)
Supplement: Supplementary file 1 [file ijms-27-09853-s001.zip › ijms-4559417-supplementary.pdf]

Supplementary Figures

Figure S1

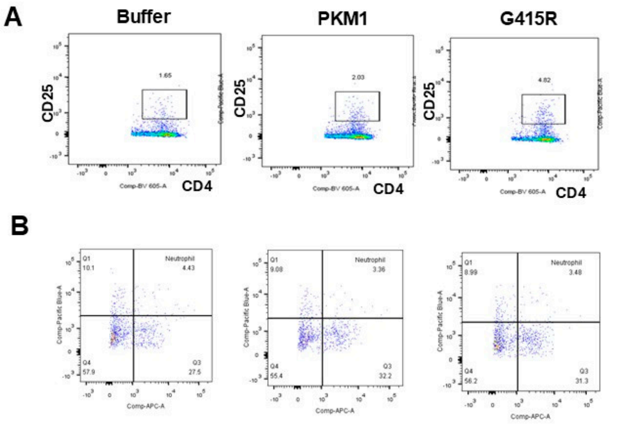

Figure S1 Examples of flow plots of FACS analysis.

(A) Examples of part of Flow plots of FACS analysis of $CD4^+$ Treg cells. **(B)** Examples of part of Flow plots of FACS analysis of neutrophils. CD206+ and CD206- macrophages in F4/80 cell populations. The gating strategies are described in method section.

Figure S2

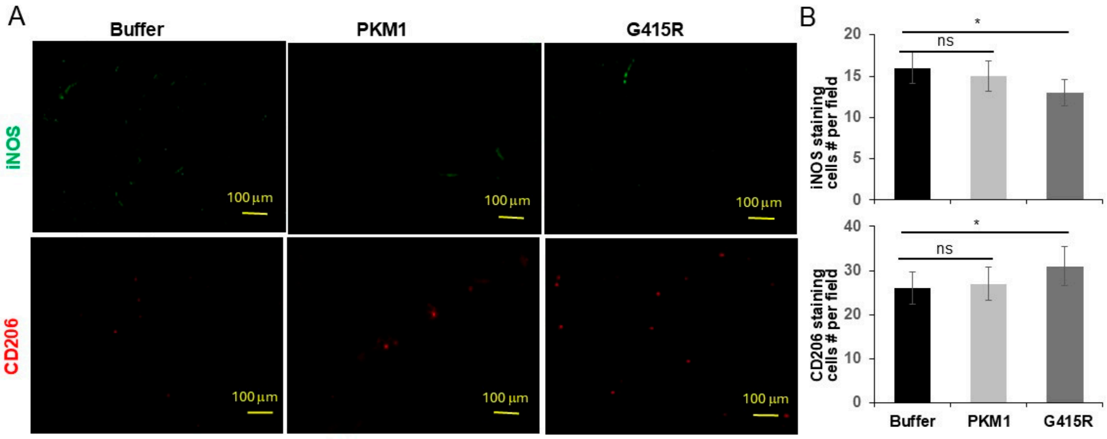

Figure S2 Macrophage M1 and M2 analyzed by IF staining,

Representative images **(A)** and quantifications **(B)** of IF staining of iNOS (M1) and CD206 (M2) macrophages in US areas of colon tissue of UC mice. The scale bars are 100 µm. Error bars in (B) represent mean ± SEM. Statistical significance was determined by one-way ANOVA. ns statistically not significant, *P < 0.05.

Figure S3

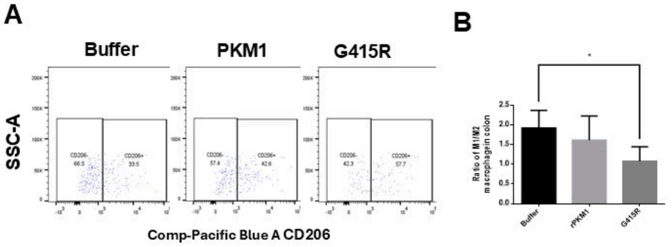

Figure S3. M1/M2 macrophage analyzed by FACS.

(A) Examples of part of Flow plots of FACS analysis of $CD206^+/CD206^-$ macrophages from $F4/80^+$ population of macrophages. **(B)** Quantifications of M1/M2 ratio of macrophages ($CD206^-/CD206^+$) in US areas of colon tissue of UC mice. Error bars in (B) represent mean ± SEM. Statistical significance was determined by one-way ANOVA, *$P < 0.05$.
